# Determinants of Decoupling Economic Output from Carbon Emission in the Transport Sector: A Comparison Study of Four Municipalities in China

**DOI:** 10.3390/ijerph16193729

**Published:** 2019-10-03

**Authors:** Qiang Wang, Shasha Wang, Rongrong Li

**Affiliations:** 1 School of Economics and Management, China University of Petroleum (East China), Qingdao, Shandong 266580, China; upc17864263755@163.com; 2 Institute for Energy Economics and Policy, China University of Petroleum (East China), Qingdao, Shandong 266580, China

**Keywords:** transport sector, Tapio decoupling model, LMDI decomposition method, driving factor

## Abstract

Quantitative analysis on decoupling between economic output, carbon emission, and the driving factors behind decoupling states can serve to make the economy grow without increasing carbon emission in China’s transport sector. In this work, we investigate the decoupling states and driving factors of decoupling states in the transport sector of China’s four municipalities (Beijing, Shanghai, Tianjin, and Chongqing) through combining the Tapio decoupling approach with the decomposition technique. The results show that (i) the decoupling state of Beijing, Shanghai, and Tianjin improved; Beijing stabilized in weak decoupling; Shanghai and Tianjin appeared to have strong decoupling, but the decoupling state of Chongqing deteriorated from decoupling to negative decoupling. (ii) The energy-saving effect was the primary contributor to decoupling in these four municipalities, promoting transport’s economic growth strongly decouple from carbon emission. The economic scale effect was not optimized enough in Chongqing, facilitating expansive coupling, and expansive negative decoupling emerged. But it had a rather positive impact on decoupling process in Beijing, Shanghai and Tianjin, promoting economic growth to weakly decouple from carbon emission. (iii) The carbon-reduction effect promoted strong decoupling, which emerged in Shanghai’s transport sector, more so than in the other three municipalities, in which weak decoupling emerged. Finally, several relevant policy recommendations were offered to promote the decoupling of carbon emission from economic growth and low-carbon transport.

## 1. Introduction

Along with the rapid development of the Chinese economy, the income of residents and the number of civil vehicles shot up, which caused the transport sector to gradually become one of major energy consumers and carbon emitters [1,2,3]. Accordingly, curbing carbon emission in the transport sector will definitely relieve carbon reduction pressure and make a great contribution to achieving the carbon reduction goal of China. Only four municipalities of China, Beijing, Shanghai, Tianjin, and Chongqing are playing the leading roles in the Chinese transport sector. These municipalities have advantageous locations, developed economies and trade, and convenient transport, which puts them in an important economic and political position. Focusing on the transport sector, their cumulative transport values have increased rapidly, with an average annual growth rate of over 10%. As the economy develops, so does carbon emission. From 2000–2016, transport carbon emission realized an incredible increase of 4.54 times in Beijing, 3.91 times in Shanghai, 1.77 times in Tianjin, and 7.75 times in Chongqing [4]. As four megacities with remarkable economic development in China, Beijing, Shanghai, Tianjin, and Chongqing have demonstrated the role to other cities of studying the decoupling state and driving factors of decoupling carbon emission from economic development in the transport sector, which is not only conducive to its own low-carbon transport construction, but provides significant reference material for other cities in China.

Hence, this paper mainly conducted relevant analyses in the following two aspects. First, it combined decoupling analysis and decomposition analysis focusing on transport sector (the new emerging major carbon emitter) of four municipalities in China. Compared with previous relevant studies, this study identified decoupling states and further identified factors influencing the decoupling process in the transport sector. As far as we know, this work is a more complete and systematical study on the decoupling relationship between carbon emission and economic growth in transport sector from a city level. Second, this paper initially conducted a systematic, comparative study between Beijing, Shanghai, Tianjin, and Chongqing, trying to figure out commonalities between them and individualities of them. The results could push these four municipalities to take lessons from each other, and attempt to take good use of advantages and avoid disadvantages from their counterparts. Besides, the results can provide empirical references to the rest cities of China, and even expanded to other countries.

This paper is organized as follows: Section 2 reviews relevant literature. Section 3 introduces Tapio’s decoupling model, the Logarithmic Mean Divisia Index (LMDI) method, and data sources. Section 4 analyses the decoupling states and driving factors of decoupling states in Beijing, Shanghai, Tianjin, and Chongqing. Section 5 summarizes the conclusions, and puts forward relevant policy recommendations, which can provide a theoretical reference for the low-carbon transport development, not only in these four municipalities, but also for the rest of the cities in China and even overseas

## 2. Literature review

### 2.1. Literature Review on Decoupling Analysis

As the second largest economy and the largest carbon emitter, China has been encountering tremendous pressure on carbon reduction due to the growing global warming issue [5,6,7]. Accordingly, that Chinese government has made numerous efforts to efficiently control energy consumption and carbon emission [8]. Furthermore, in response to the Paris Agreement, China committed to decrease carbon intensity by 60%–65% by 2030 relative to the 2005 level and reach a peak of carbon emission by 2030 [9,10]. However, China is experiencing rapid urbanization and industrialization marked by a dramatically growing economic output, and immerse energy consumption and carbon emissions. There is no chance to sacrifice economic growth in return for environmental improvement. Hence, how to emit less or even zero carbon dioxide while keeping the economy blossoming is deemed the key to fulfill the commitment of China about carbon reduction. To do that efficiently, decoupling carbon emission from economic growth shall be taken into consideration.

Numerous scholars who explored the decoupling relationship between economic growth and carbon emission in China mainly focused on two levels: industry and region. From the perspective of region, some scholars regarded China as a whole when investigating decoupling economic growth from carbon emission, like Zhang [11] and Riti et al. [12]. Wang et al. [13] took full consideration of urbanization and industrialization, in that way investigated the decoupling state and driving factors of China and India. Zhou et al. [14] investigated decoupling relationship between carbon emission and economic growth in eight major regions of China in 1996–2012. The results suggested most regions emerged with weak decoupling. Wang et al. [15] compared carbon performance and decoupling performance between China and the United States. The results showed China was more expansive coupling and had weak decoupling, while the United States was more in weak decoupling and decoupled strongly, apparently better than China. Other scholars preferred to conduct a decoupling analysis on the provincial level; for instance, the developed Jiangsu province [16,17,18], the first that implemented reform and opening-up to the West, and the number one economic province, Guangdong [19,20]. Wang and Jiang [21] identified decoupling state and further explored factors influencing decoupling process. They found China was dominated by weak decoupling. Wang and Yang [22] identified decoupling relationship between industrial economic output and carbon emissions in the Beijing–Tianjin–Hebei region through Tapio decoupling and the LMDI decomposition method. They found weak decoupling was the main decoupling state for the majority of years. Zhang et al. [23] studied decoupling industrial growth from carbon emission in Xinjiang. They found that the decoupling state was not stable enough, changing from negative decoupling to weak decoupling, then to negative decoupling. As for city level, relevant studies are relative rarer. Yu et al. [24] investigated decoupling of economic growth from environmental pressure (one of indicators is carbon emission) from 1999 to 2010 in Chongqing. The results indicated that technological change took precedence over the rest of the factors driving decoupling. Wang et al. [25] performed a comparative analysis between Beijing and Shanghai from a sector-level. They found that the decoupling performance of Beijing’s industry was better than that of Shanghai, and the Shanghai’s transport sector required more decoupling efforts. Li et al. [26] conducted a decoupling and uncertainty analysis in 29 cities of China’s Central Plains urban agglomeration. From the perspective of industry, Ren et al. [27] and Wan et al. [28] studied decoupling levels in Chinese manufacturing/equipment manufacturing industry. Tang et al. [29] explored the tourism industry during 1990–2012 and found that decoupling states alternated in negative and weak decoupling. Zhu and Li [30] researched the decoupling of carbon from the economy in the Beijing–Tianjin–Hebei Area in transport sector. Li et al. [2] examined decoupling relationship in transport sector from provincial level using Tapio decoupling model. Wu et al. [31] and Lu et al. [32], respectively, conducted a decoupling analysis in the construction industry; the former from national and provincial levels during 2005–2015, and the latter only from the national level during 1994–2012. Besides, some other scholars paid attention to other industries, like Lin and Liu in heavy industry [33], Jiang et al. [34] in six major Chinese sectors, and so on.

The Chinese government formulated carbon reduction policies and regulations on a national level and usually implemented them on provincial and urban levels [35,36]. With Chinese rapid urbanization and industrialization, the scale of city and industry is bound to be constantly expand, which will cause more people to flow into cities and much more energy to be consumed and carbon emitted. As a result, cities will play a significant role in carbon reduction. The transport sector is the basis of the national economy and social development [37,38]. As per capita disposable income and civil vehicles increasing year by year, the transport sector has become the new main energy consumer and carbon emitter. To decouple carbon emissions from transport’s economic growth at the city level will not only be conductive to low-carbon transport and low-carbon city construction, but also facilitate effective and specific implementation of carbon reduction policies and regulations at city level, so as to achieve the national carbon reduction goal as soon as possible.

### 2.2. Literature Review on Decomposition Analysis

However, only conducting decoupling analysis failed to capture environmental externalities and reveal the inner mechanism of decoupling [39,40]. Hence, we went a step forward to combine a decomposition analysis with decoupling analysis to figure out what drives transport’s carbon emission decouple from economic growth in these four municipalities. 

Actually, a large number of decomposition analysis have been done in transport sectors of Beijing, Shanghai, Tianjin, and Chongqing, but the majority of them preferred to investigate factors influencing carbon emission rather than the decoupling process in transport sector [41]. Regarding to Beijing, Wang and Liu [42] studied influence of individual travel behavior on urban transport carbon emission from 2000 to 2011. The results indicated that transport intensity as well as the emission coefficient significantly influenced carbon reduction. Ma et al. [43] investigated transport carbon emissions and made an estimation of carbon emission from the disaggregate level. Fan and Lei [44] applied the multivariate, generalized Fisher index model to study influencing factors on transport carbon emission from 1995 to 2012. They discovered transport intensity played a negative role, while energy structure played a positive role in reducing carbon emission. Regarding to Shanghai, Wang et al. [45] applied the LMDI method to identify factors driving carbon emission in the passenger transport sector. Wu et al. [46] decomposed carbon emissions in transport sector by the LMDI method, and they found energy intensity and energy structure effect played important roles in reducing carbon emissions/intensity. Moreover, relevant decomposition analyses about transport sector in Tianjin and Chongqing were rather rarer. 

To the great extent of our knowledge, our work conducting a combination of decoupling analysis and decomposition analysis in the transport sector of Beijing, Shanghai, Tianjin, and Chongqing shall be considered as the first complete and systematical attempt to identify decoupling state between carbon emission and economic growth and further explore influencing factors of decoupling process in transport sector at a city level in China.

## 3. Materials and Methods

### 3.1. Calculation of Carbon Emission in Transport Sector

We selected two variables, total carbon emission (CO_2_), and transport’s added value (GTP), to study the decoupling relationship between carbon emissions and economic development in Beijing, Shanghai, Tianjin, and Chongqing.

Since China’s statistical agencies do not separately assess the carbon emissions of the transport sector, transport carbon emission needed to be calculated based on the energy consumption of transport sector. According to China’s current statistical caliber, in the statistics of energy consumption of individual cities by sector, transport, warehousing, and postal sectors are put into one sector (hereinafter referring to transport), which mainly consumes fossil energy, such as raw coal, coke, fuel oil, gasoline, diesel, kerosene, and natural gas. Referring to the energy consumption carbon emission calculation method described in the IPCC National Greenhouse Gas Inventory [47], the carbon emissions of the transport sectors in Beijing, Shanghai, Tianjin, and Chongqing are as follows:

(1) $$CO_{2}=\sum_{i}E_{i}\times T_{i}\times e_{i}\times O_{i}$$

where *i* indicates the energy type; and *E, T, e*, and *O* respectively represent the total energy consumption, energy conversion coefficient, carbon emission factor, and carbon oxidation rate. More detailed data are shown in the following Table 1.

### 3.2. Carbon Decoupling Model

Decoupling is widely appreciated as a physical theory used to describe the reduction or disappearance of the correlation between two or more physical quantities. In the analysis of the relationship between economic development and environmental quality, decoupling is defined as the break of a coupling relationship between the two variables. This paper uses Tapio’s [48] decoupling elastic model to study the relationship between carbon emission and economic development in Beijing, Shanghai, Tianjin, and Chongqing. The formula is as follows:

(2) $$t=\frac{(CO_{2}{}^{\alpha }-CO_{2}{}^{0})/CO_{2}{}^{0}}{\left(GTP^{\alpha }-GTP^{0}\right)/GTP^{0}}=\frac{∆CO_{2}/CO_{2}{}^{0}}{∆GTP/GTP^{0}}$$

where t indicates the decoupling elastic coefficient of carbon emission and economic development in the transport sector; α and 0 indicate the targeted year and base year, respectively.$\;∆CO_{2}/CO_{2}{}^{0}$ represents the carbon emission growth rate of the transport sector (hereinafter indicated by %*CO_2_*). $∆GTP/GTP^{0}$ represents the economic growth rate of transport sector (hereinafter indicated by %GTP).

**Figure 1:**
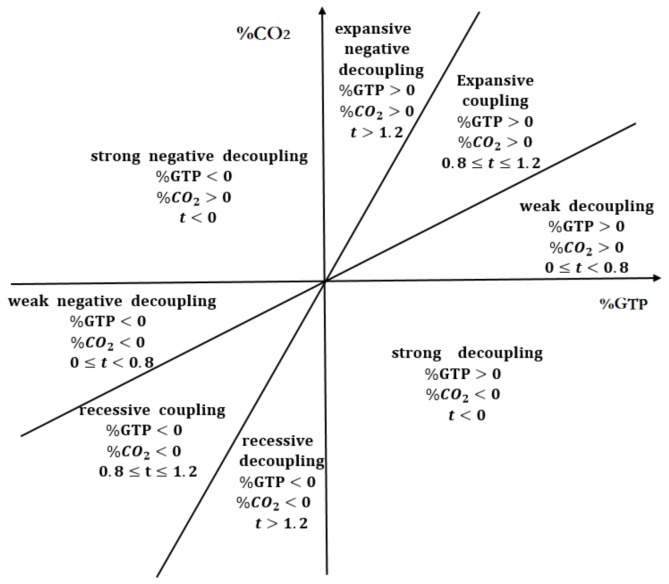
Tapio decoupling model of carbon emission and economic growth.

Based on Tapio’s division of decoupling coefficient between carbon emissions and economic development, we drew a picture of a decoupling model between carbon emissions and economic development in transport sector, as shown in Figure 1.

The decoupling state can be divided into three categories: decoupling, negative decoupling, and coupling, and then subdivided into strong decoupling, weak decoupling, recessive decoupling, strong negative decoupling, weak negative decoupling, expanding negative decoupling, expansive coupling, and recessive coupling. Among them, strong decoupling is the most ideal state, indicating that the transport’s added value continues to increase (%GTP > 0), while the total amount of carbon emission decreasing (%CO2 < 0); strong negative decoupling is the most unfavorable state, indicating that the transport’s added value is reducing (%GTP < 0), while the total amount of carbon emission is increasing (%CO2 > 0).

In the realistic study of the relationship between carbon emission and economic development, there exists less extreme cases of strong decoupling and strong negative decoupling; the expansive negative decoupling and weak decoupling are far more common, and the decoupling states are dynamic. Deepening analysis of decoupling states and driving factors have a positive impact on the development of a low-carbon economy.

### 3.3. Using the LMDI Method on the Driving Factors of Transport Carbon Emission

The general decomposition method consists of structural decomposition analysis (SDA) [49] and index decomposition analysis (IDA) [50]. SDA has mainly been scientifically applied on national or regional scales [51]. But the input–output table needed in SDA decomposition is hard to obtain, which leads to the application of SDA being limited. Regarding to IDA, it requires less data, so it is more widely used in the field of energy and environmental study [52]. In addition, Ang compared various index decomposition analysis methods, and drew the conclusion that the LMDI is the preferred method [53], because it leaves no residuals and handles a zero-value perfectly [21,52]. Therefore, when conducting decoupling and decomposition analysis, LMDI was developed in this paper.

On the basis of Kaya’s identity [54], this paper applies LMDI method, which is without residuals, to decompose the total carbon emissions of Beijing, Shanghai, Tianjin, and Chongqing as follows:

(3) $$CO_{2}=\frac{CO_{2}}{E}\times \frac{E}{GTP}\times \frac{GTP}{GDP}\times \frac{GDP}{P}\times P$$

where *P* indicates the total population. Let $r=\frac{CO_{2}}{E}$ indicates the energy carbon emission intensity; $s=\frac{E}{GTP}$ indicates the energy consumption intensity of per unit added value of transport sector; $d=\frac{GTP}{GDP}$ indicates the share of transport’s added value to gross domestic product (GDP); $f=\frac{GDP}{P}$ indicates per-capita income of the specific municipality. Therefore, the decomposition model of total carbon emission can be further described as:

(4) $$CO_{2}=r\times s\times d\times f\times P$$

The LMDI decomposition method consists of a multiplicative form and an addition form. However, they are the same in essence, and easy to convert to each other. Therefore, this paper uses the LMDI addition-decomposition method to decompose the change of total carbon emission from the base phase (indicated by 0) to the targeted phase (indicated by α) as follows:

(5) $$∆CO_{2}=CO_{2}^{\alpha }-CO_{2}^{0}\;=r^{\alpha }\times s^{\alpha }\times d^{\alpha }\times f^{\alpha }\times P^{\alpha }-r^{0}\times s^{0}\times d^{0}\times f^{0}\times P^{0}\;=∆C_{r}+∆C_{s}+∆C_{d}+∆C_{f}+∆C^{P}$$

(6) $$∆C_{r}=\left(\frac{CO_{2}^{\alpha }-CO_{2}^{0}}{lnCO_{2}^{\alpha }-lnCO_{2}^{0}}\right)\times ln\frac{r^{\alpha }}{r^{0}}$$

(7) $$∆C_{s}=\left(\frac{CO_{2}^{\alpha }-CO_{2}^{0}}{lnCO_{2}^{\alpha }-lnCO_{2}^{0}}\right)\times ln\frac{s^{\alpha }}{s^{0}}$$

(8) $$∆C_{d}=\left(\frac{CO_{2}^{\alpha }-CO_{2}^{0}}{lnCO_{2}^{\alpha }-lnCO_{2}^{0}}\right)\times ln\frac{d^{\alpha }}{d^{0}}$$

(9) $$∆C_{f}=\left(\frac{CO_{2}^{\alpha }-CO_{2}^{0}}{lnCO_{2}^{\alpha }-lnCO_{2}^{0}}\right)\times ln\frac{f^{\alpha }}{f^{0}}$$

(10) $$∆C_{P}=\left(\frac{CO_{2}^{\alpha }-CO_{2}^{0}}{lnCO_{2}^{\alpha }-lnCO_{2}^{0}}\right)\times ln\frac{P^{\alpha }}{P^{0}}$$

Among them, $∆CO_{2}$ represents the total carbon emission change of the transport sector. $∆C_{r},∆C_{s},∆C_{d},∆C_{f,}\mathrm{and}\;∆C_{P},$ respectively, indicate the emission-reduction effect, energy-saving effect, transport share effect, economic scale effect, and population scale effect of the transport sector.

Combining Equations (1) and (5), on the basis of the decoupling model of carbon emission and economic development in the transport sector, the decomposition of the decoupling model can be further obtained, through LMDI decomposition method:

(11) $$t=\frac{∆CO_{2}/CO_{2}}{∆GTP/GTP}=∆CO_{2}\times \frac{GTP}{CO_{2}\times ∆GTP}\;=\left(∆C_{r}+∆C_{s}+∆C_{d}+∆C_{f}+∆C_{P}\right)\;\times \frac{GTP}{CO_{2}\times ∆GTP}\;=\frac{∆C_{r}/CO_{2}}{∆GTP/GTP}+\frac{∆C_{s}/CO_{2}}{∆GTP/GTP}+\frac{∆C_{d}/CO_{2}}{∆GTP/GTP}+\frac{∆C_{f}/CO_{2}}{∆GTP/GTP}+\frac{∆C_{P}/CO_{2}}{∆GTP/GTP}\;=t_{r}+t_{s}+t_{d}+t_{f}+t_{P}$$

In the formula, $t_{r},t_{s},t_{d},t_{f},t_{P}$ respectively represent the decoupling elastic coefficient of the emission-reduction effect, energy-saving effect, transport share effect, economic scale effect, and population scale effect of transport sector.

### 3.4. Data Sources

This paper calculates the total amount of carbon emission based on the energy consumption of the transport sector. The relevant energy consumption and energy conversion coefficients are from the China Energy Statistical Yearbook 2000–2016 [55]. This paper selects 2000–2015 [56] as the research period. The added value and total population of the transport sector in Beijing, Shanghai, Tianjin, and Chongqing are derived from the China Statistical Yearbook 2000–2016. In order to eliminate the influence of inflation factors, this paper uses the GDP deflator to adjust the value added of transport sector to the 2000 price level.

## 4. Empirical Results and Analysis

### 4.1. Study Overview 

Prior to investigating decoupling economic growth from carbon emissions in the transport sector of Beijing, Shanghai, Tianjin, and Chongqing, it is necessary to take a look at the situation of transport’s carbon emissions and economic growth in these four municipalities, which will significantly facilitate the conduction of further decoupling analysis and decomposition analysis.

#### 4.1.1. Beijing City

**Figure 2:**
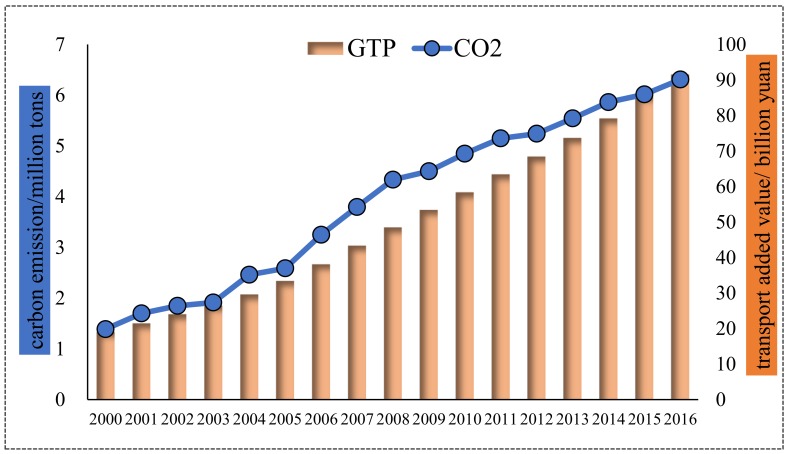
Transport’s carbon emissions and economic output for Beijing.

As shown in Figure 2, both carbon emissions and added value of the transport sector constantly increased in Beijing from 2000–2016. Focusing on transport’s added value, it stably increased from 19.01 billion yuan in 2000 to 91.57 billion yuan in 2016, with an annual growth rate of 10.32%. As the economic output grew drastically, so did energy consumption and carbon emission. Transport carbon emissions appeared to have an upward trend and the growth rate obviously accelerated in 2005–2008, which was in the period of the Eleventh Five-year Plan (FYP); then, it slightly declined and maintained a gentle growth rate. In general, transport’s carbon emissions increased from 1.39 million tons (Mt) in 2000 to 6.31 Mt in 2016, increasing by 4.92 Mt, with an annual growth rate of 9.92%, a little lower than that of added value. Generally speaking, the economic growth of Beijing’s transport sector was accompanied by heavy carbon emission, but fortunately, transport’s economic output grew a little faster than carbon emissions, which indicated that Beijing’s energy-saving and emission reduction measures have achieved initial results.

#### 4.1.2. Shanghai City

**Figure 3:**
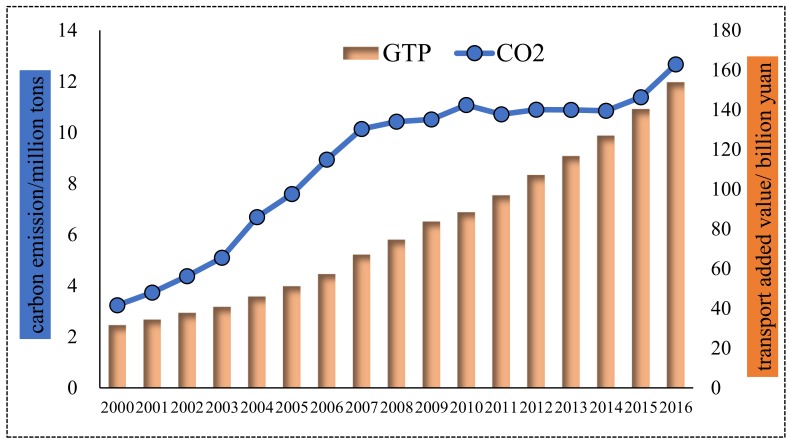
Transport carbon emission and economic output for Shanghai.

The changes of transport’s added value and the carbon emissions of Shanghai are depicted in Figure 3. From Figure 3, it can be seen than carbon emissions of Shanghai’s transport sector experienced three stages: it smoothly increased in 2000–2007, maintained stability and slightly decreased in 2007–2014, and rapidly increased in 2014–2016. That indicates that Shanghai’s transport sector gradually curbed carbon-emission’s increase recently. As a whole, carbon emission increased from 3.24 Mt in 2000 to 12.66 Mt in 2016 with an annual growth rate of 8.9%, a bit lower than that of Beijing’s transport sector’s carbon emission. Regarding transport’s added value to Shanghai, it produced 5.7 billion yuan per year in the tenth and eleventh FYPs (2000–2010) and 12.6 billion yuan per year in 2011–2016. The transport sector in Shanghai was growing with an incredible speed and produced more economic output. Moreover, the annual growth rate of added value (10.43%) was higher than that of carbon emission (8.9%) and carbon emission roughly maintained stability since 2007 (except 2014–2016), which uncovered that Shanghai’s energy-saving and emission-reduction technologies are increasingly mature, and can effectively curb the increase of transport carbon emission at the time of economic development.

#### 4.1.3. Tianjin City

**Figure 4:**
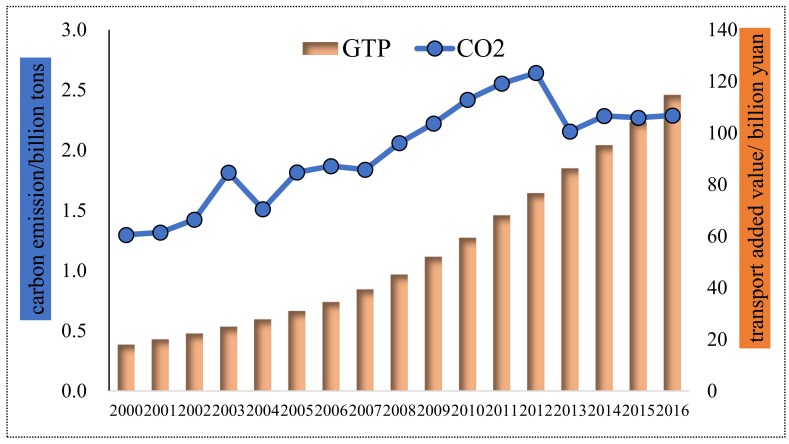
Transport carbon emissions and economic output for Tianjin.

From Figure 4, transport’s added value to Tianjin increased year by year, from 17.88 billion yuan in 2000 to 114.82 billion yuan in 2016 with a pretty high annual growth rate of 12.32%, higher than the rest three municipalities. As for transport carbon emission, it increased with fluctuation in 2000–2012 and decreased and tended to maintain stable in 2012–2016. Moreover, transport’s carbon emissions in Tianjin increased by 0.99 Mt overall and with an annual growth rate of 3.61%, far lower than the other three municipalities. To our surprise, from the point of the total amount, transport’s added value to Tianjin ranked second place, while carbon emission rather ranked the last place. Furthermore, though transport carbon emission and the added value of Tianjin increased in the study period, the latter grew far faster than the former. It demonstrated that Tianjin has already implemented effective carbon reduction policies and measures and has impressively achieved weakened the synchronous increase of transport’s added value and carbon emissions in Tianjin.

#### 4.1.4. Chongqing City

**Figure 5:**
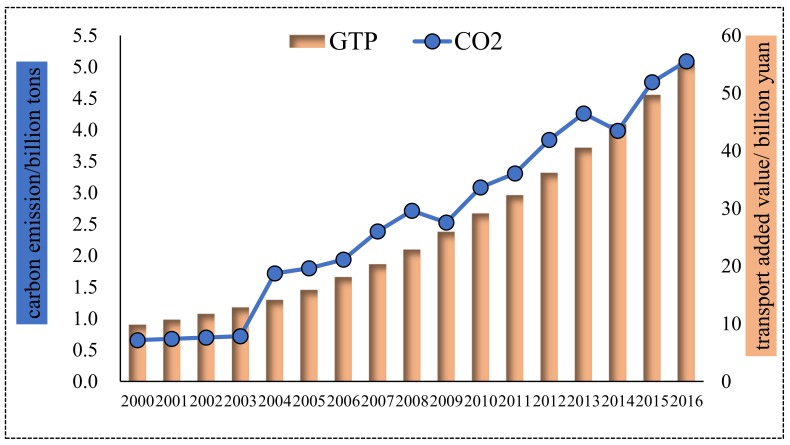
Transport’s carbon emissions and economic output for Chongqing.

As evidently depicted in Figure 5, transport’s carbon emissions experienced two stages: relatively stable in 2000–2003 and increased with fluctuation in 2003–2016. Overall, carbon emission increased from 0.66 Mt in 2000 to 5.09 Mt in 2016, increased by 4.43 Mt with a quite high annual growth rate of 13.66%. For transport’s added value, it presented a continuous increasing trend, from 9.82 billion yuan to 55.16 billion yuan in the period of 2000–2016, with a rather high annual growth rate of 11.39%. What deserves to be highlighted is that though transport’s carbon emissions and added value grew with high speeds, the former grew faster than the latter. It uncovered that transport development is closely connected with energy consumption and carbon emission, which will hinder carbon reduction and low-carbon economy construction, and require more effort to improve the poor situation.

Overall, transport’s added value all increased in 2000–2016 in the four municipalities with a high annual growth rate. But carbon emission performed differently in these four municipalities. Carbon emission in Beijing and Chongqing appeared to have a constantly increasing tread, but tended to be stable and even decreasing in Shanghai and Tianjin, indicating carbon emission has been effectively controlled in these two municipalities. Different carbon emission performance and added value performance, would lead to different decoupling performance. After discussing the situation of transport’s added value and carbon emission in these four municipalities, we will analyze the decoupling relationship between transport’s added value and carbon emissions for the next step.

### 4.2. Analysis on the Decoupling State of the Transport Sector

In order to identify the relationship between the carbon emission and economic growth of the transport sector, on the basis of data availability, the Tapio decoupling analysis was performed in 2000–2016 with specific analyses in these four municipalities.

#### 4.2.1. Beijing City

**Figure 6:**
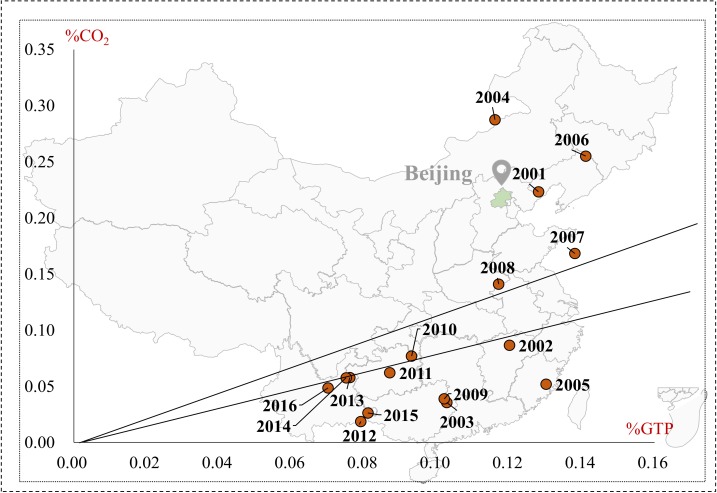
The decoupling states of carbon emission and the economic growth in Beijing’s transport sector.

Decoupling states between transport carbon emission and economic growth in Beijing presented an improving trend from 2000 to 2016 (see Figure 6). During the period of 2000–2008, Beijing was in expansive negative decoupling and weak decoupling states, indicating transport’s carbon emissions and economic growth had a close connection. During the period of 2009–2016, Beijing stabilized in a weak decoupling state (except 2009–2010 in expansive coupling), which demonstrated that Beijing’s transport sector gradually weakened the connection between carbon emission and economic growth and promoted transport’s economic growth to weakly decouple from carbon emission. On the whole, the decoupling state of Beijing’s transport sector shifted from negative decoupling to decoupling, and finally, maintained a stable weak decoupling, indicating that policies and measurements of energy conservation and constructing comprehensive transport were in force, which echoed with the results of Zhao et al [57], who found transport’s decoupling state improving from negative decoupling to decoupling. However, Beijing still has much room to improve its decoupling relationship between transport carbon emissions and economic growth, which requires more effort made to accelerate transport’s economic growth to strongly decouple from carbon emission.

#### 4.2.2. Shanghai City

**Figure 7:**
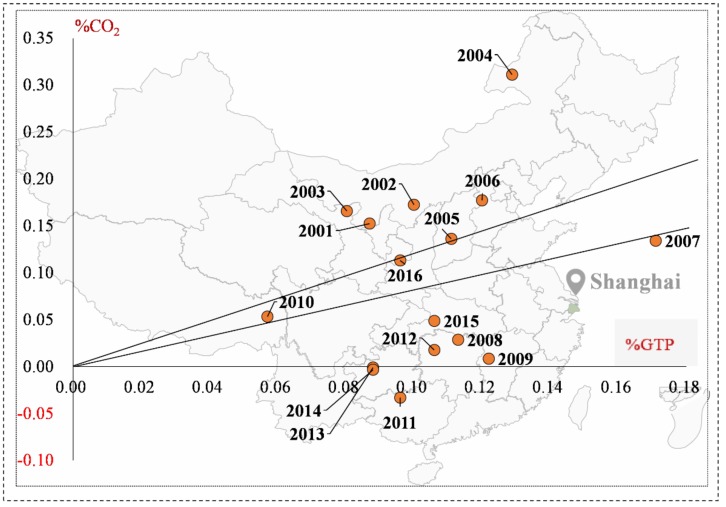
The decoupling states of carbon emission and economic growth in Shanghai’s transport sector.

The decoupling state between transport’s carbon emissions and economic growth in Shanghai was described in Figure 7. Shanghai appeared to have expansive negative decoupling in 2000–2006, with a high average decoupling index of 1.78, which manifested that transport carbon emissions grew far faster than economic growth. The study of Wang et al. [25] also confirmed our findings that Shanghai’s transport sector wad rather bad in the first stage. From 2006 to 2009, shanghai appeared with weak decoupling, but the decoupling state improved a lot compared with the former period. With the heavy influence of global economic crisis, the decoupling state of Shanghai’s transport sector changed violently in 2009–2016, emerging in three decoupling states: expansive coupling, weak decoupling, and strong decoupling. What is worth highlighting is that Shanghai appears to have the best decoupling state, strong decoupling, and has great potential to promote transport’s economic growth to strongly decouple from carbon emissions.

#### 4.2.3. Tianjin city

**Figure 8:**
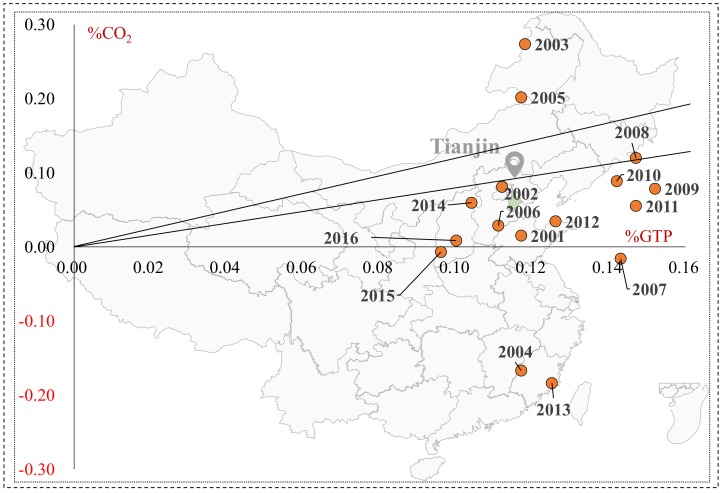
The decoupling states of carbon emission and economic growth in Tianjin’s transport sector.

As shown in Figure 8, Tianjin was in weak decoupling for the majority of years. Specifically, it appeared to have expansive negative decoupling, expansive coupling, weak decoupling, and strong decoupling in 2000–2008. For the remaining years, Tianjin presented weak decoupling in 2008–2012, 2013–2014, and 20115–2016, but strong decoupling in 2012–2013 and 2014–2015. Overall, the decoupling state between transport’s carbon emissions and economic growth in Tianjin was stabilized in weak decoupling and appeared to have the trend of strong decoupling.

#### 4.2.4. Chongqing City 

**Figure 9:**
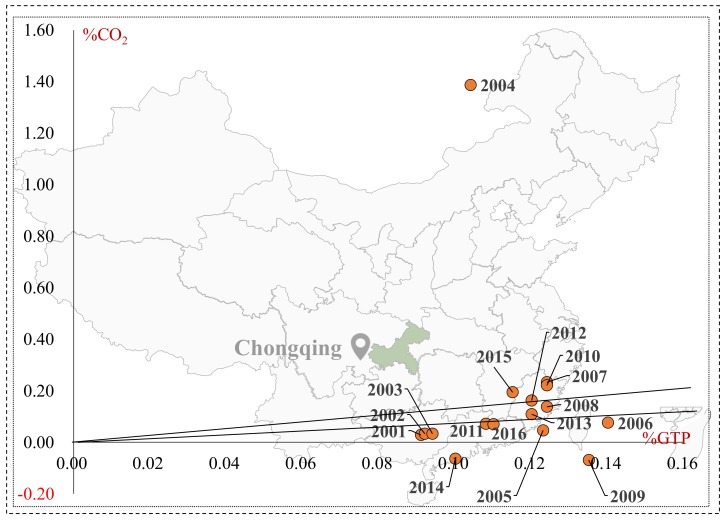
The decoupling states of carbon emission and economic growth in Chongqing’s transport sector.

As in Figure 9, it can be seen Chongqing appeared four decoupling states in 2000–2016: expansive negative decoupling, expansive coupling, weak decoupling, and strong decoupling. Transport’s economic growth weakly decoupled from carbon emission in 2000–2006 (except 2003–2004). For the remaining years, though Chongqing appeared to have strong decoupling in 2008–2009 and 2013–2014, it was dominated by a worse decoupling state, expansive negative decoupling. In general, the decoupling relationship between transport carbon emission and economic growth in Chongqing deteriorated with time going by, which can be attributed to the rapid transport economic development, which required more energy consumption and emitted more carbon.

Regarding the decoupling relationship between transport’s carbon emissions and economic growth, Beijing, Shanghai, and Tianjin all improved a lot. Beijing stabilized in a weak decoupling state, while Shanghai and Tianjin appeared to have the trend of strong decoupling. According to the study by Jiang et al. [34], China’s transport sector was dominated by expansive coupling, which indicated that transport sectors of Beijing, Shanghai, and Tianjin have reached a better decoupling situation compared with the whole China. However, to our surprise, the decoupling state of the Chongqing transport sector deteriorated in 2000–2016, varying from stable weak decoupling to expansive negative decoupling and expansive coupling.

### 4.3. Analysis of the Driving Factors of Carbon Emission Decoupling in the Transport Sector

After obtaining the decoupling states of the four municipalities in China (Beijing, Shanghai, Tianjin, and Chongqing), we took a step forward to investigate factors influencing decoupling state. In order to do that effectively, we decomposed total carbon emission into five influencing factors using the LMDI decomposition method: the emission-reduction effect, energy-saving effect, transport share effect, economic scale effect, and population scale effect, and introduced them into the Tapio decoupling model.

#### 4.3.1. Beijing City

The impact of all individual factors on decoupling in Beijing is displayed in Table 2. Generally speaking, Beijing’s transport sector improving from expansive negative decoupling to stable weak decoupling. The carbon-reduction effect promoted transport’s economic growth to weakly decouple from carbon emission for almost years. Moreover, the carbon-reduction effect accelerated a strong decoupling beginning in 2011, which demonstrated Beijing’s transport sector greatly optimized energy consumption. The raw coal consumption ratio in total energy consumption decreased from 12.62% to 1.04%; natural gas increased from 0 to the highest proportion, 3.95%, in 2000–2016. Regarding the energy-saving effect, its impact on decoupling was not optimal in 2000–2008, appearing with not only strong decoupling, but also weak decoupling and even expansive negative decoupling. Fortunately, the energy-saving effect stably facilitated a strong decoupling process in Beijing’s transport sector throughout 2008–2016. The other three factors typically accelerated transport’s economic growth in weakly decoupling from carbon emissions, but there also existed differences among them. Transport’s share boosted the appearance of a strong decoupling state in 2002–2004, 2008–2010, and 2012–2013. The economic scale effect appeared to have expansive coupling in 2003–2004 and 2015–2016. A population scale effect promoted transport’s economic growth to weakly decouple from carbon emission constantly and prompted the connection between economic growth and carbon emissions to weaken with time going by. 

#### 4.3.2. Shanghai City

As shown in Table 3, Shanghai’s transport sector improved from expansive negative decoupling to weak decoupling and appeared with the trend of strong decoupling. The carbon-reduction effect almost accelerated weak decoupling prior to 2007 and strong decoupling for the remaining years. It uncovered that Shanghai’s transport sector positively shifted from carbon intensive energy to low-carbon energy under the umbrella of energy transformation; for example, the ration of fuel oil decreased from 64.93% to 37.49% in 2000–2016, raw coal decreased from 3.67% in 2001 to 0.05% in 2016, and natural gas increased from 0 to the highest ratio of 0.43% in 2000–2016. The energy-saving effect significantly promoted transport’s economic growth to strongly decouple from carbon emission, except in a few years. Especially in 2003–2004, Shanghai’s transport sector appeared with expansive coupling and expansive negative decoupling. Since then, China joined the World Trade Organization (WTO), so the transport sector rapidly developed, without enough attention paid to energy-saving and carbon-reduction [58,59]. The transport share changed from strong decoupling to weak decoupling. The economic scale effect had a better impact on the decoupling situation of Shanghai’s transport sector, changing from expansive coupling to weak decoupling. Like Beijing’s transport sector, the population scale effect also stably promoted Shanghai transport’s economic growth to weakly decouple from carbon emission and the impact is gradually strengthening.

#### 4.3.3. Tianjin City

Table 4 clearly displays the impact of all factors on the decoupling process of Tianjin. The carbon-reduction effect promoted the appearance of weak decoupling and even strong decoupling in Tianjin transport sector. The energy-saving effect continuously promoted transport’s economic growth to strongly decouple from carbon emission from 2000–2016 (except 2002–2003 in expansive negative decoupling and 2004–2005 in weak decoupling), which was the best state of carbon emission and economic growth. It demonstrated Tianjin possessed a better energy use efficiency than the others, which could produce more economic output yet with less energy consumption. Transport share promoted strong decoupling in 2000–2012 and weak decoupling in 2012–2016. Though the impact of transport share deteriorated since 2012, the average decoupling indexes (0.04) were extremely small, indicating that transport’s economy grew far faster than carbon emissions in the transport sector. The economic scale effect had a positive impact on decoupling, from expansive coupling to weak decoupling. As for population, which was like Beijing and Shanghai’s transport sector, it stably promoted transport’s economic growth to weakly decouple from carbon emission.

#### 4.3.4. Chongqing City

From Table 5, it can be seen that decoupling situation of Chongqing’s transport sector was not optimal enough, appearing with expansive negative decoupling and expansive coupling for years. The carbon-reduction effect drove transport’s economic growth to weakly decouple from carbon emission for some years. Regarding the energy-saving effect, it appeared to strongly decouple transport’s carbon emission from economic growth in 2000–2006 (except 2003–2004 in expansive negative decoupling). However, it alternated between weak decoupling and strong decoupling in 2006–2016, indicating the energy intensity of Chongqing’s transport sector still needed make as much effort as possible to improve energy intensity. The transport share effect promoted the emergence of strong decoupling, which indicated a reduction in the ratio of transport’s economic output in GDP would make positive impact on the decoupling process. Different from the above three municipalities, the economic scale effect exerted a negative impact on decoupling of Chongqing’s transport sector, facilitating the emergence of expansive coupling and even expansive negative decoupling. Therefore, economic scale was still the main inhibitor on decoupling and needed to be paid much more attention. As for the population scale effect, its impact on decoupling was weakening, from strong decoupling to weak decoupling, indicating that carbon emission increased drastically when the population scale expanded.

## 5. Discussion

Overall, transport’s added value of the four municipalities (Beijing, Shanghai, Tianjin, and Chongqing) all constantly increased from 2000 to 2016. When it comes to a comparison among them about transport’s added value, from the perspective of total amount, Shanghai always ranked the first and Chongqing always ranked the last, while Tianjin surpassed Beijing and ranked second since 2010; from the perspective of annual growth rate: Tianjin (12.32%) > Chongqing (11.39) > Shanghai (10.43%) > Beijing (10.32%). Regarding transport’s carbon emissions, Beijing and Chongqing increased all the time, while Shanghai has maintained stability since 2007; Tianjin initially achieved significant carbon reduction. As for transport’s total carbon emission, Shanghai ranked the first, followed by Beijing, while Chongqing surpassed Tianjin to become the third largest carbon emitter among these four municipalities in 2006. In addition, from the point of the annual growth rate of carbon emission: Chongqing (13.66%) > Beijing (9.92%) > Shanghai (8.9%) > Tianjin (3.61%). What Shanghai and Tianjin need to do next is make efforts to lower carbon emission, while Beijing and Chongqing shall pay much more attention to curb carbon emissions. Moreover, in order to fulfil Chinese commitment on carbon reduction by 2030, energy optimization shall be taken into consideration, increasing ratios of clean and renewable energies (such as wind power and solar power) and decreasing ratios of carbon-intensive energies.

With respect to decoupling state, there only appeared four decoupling states, expansive negative decoupling, expansive coupling, weak decoupling, and strong decoupling, in these four municipalities. Beijing had, in the periods measured, expansive negative decoupling, weak decoupling, weak decoupling, and finally, stabilized in weak decoupling; Shanghai experienced a transition: expansive negative decoupling → weak decoupling → expansive coupling, weak decoupling, and strong decoupling. Though decoupling state of Shanghai’s transport sector was not stable recently, it appeared to have a pleasantly improving trend; the decoupling state of Tianjin transport sector was better than the other three municipalities. It appeared with weak decoupling for almost all years and showed the obvious trend of strong decoupling. Beijing, shanghai, and Tianjin have already gotten a relatively good decoupling state, but they still had a long way to achieve strong decoupling. Therefore, what they need to do is exert more efforts to emit less carbon emission in future, transport-related economic development. As a result, they should focus on stabilizing the strongly decoupling of carbon emission from economic development in the transport sector. Different from the above three improving municipalities, Chongqing’s transport sector deteriorated a lot from weak decoupling to expansive coupling, and to expansive negative decoupling. For Chongqing, there existed great potential to mitigate carbon emission in the course of economic development and promote carbon emission to strongly decouple from economic growth in the transport sector. Hence, in the process of low-carbon alternatives’ developments, efforts should be made to promote the stable decoupling between carbon emission and economic development in the transport sector to try to achieve strong decoupling.

As for all individual factors, the carbon-reduction effect significantly promoted transport’s economic growth to weakly decouple from carbon emission, but its impact on the decoupling of Shanghai and Tianjin was better than that of Beijing and Chongqing, because carbon-reduction effect gradually promoted strong decoupling in Shanghai and Tianjin’s transport sectors. The energy-saving effect obviously promoted strong decoupling in the four municipalities, but the positive impact of the energy-saving effect of Chongqing started weakening recently. The transport share effect promoted economic growth to strongly decouple from carbon emission among the four municipalities, especially in Tianjin and Chongqing. The economic scale effect mainly promoted weak decoupling between carbon emission and economic growth in Beijing, Shanghai, and Tianjin transport sector’s, but in Chongqing, it showed expansive coupling and expansive negative decoupling. It demonstrated that Beijing, Shanghai, and Tianjin had already effectively curbed carbon emissions’ increases with economic growth, but in Chongqing, the economic scale effect was still the primary inhibitor of the decoupling process. The population scale effect stably promoted weak decoupling, indicating carbon increased quickly with population scale expansion.

## 6. Conclusions and Policy Recommendations

As the economy developed, more energy was consumed and more carbon was emitted in the transport sector, a newly emerging, major carbon emitter. In the world of promoting economic development, relieving carbon reduction pressure needs to be paid more attention in relation to the relationship between carbon emission and economic growth, trying to emit less carbon without harming economic growth [60]. Hence, we selected Beijing, Shanghai, Tianjin, and Chongqing, the only four municipalities to identify with a decoupling state for the first step, and then investigated the factors driving the decoupling process from 2000–2016. Through our complete and comprehensive study, we found the situation of Tianjin’s transport sector was better than that of the other three municipalities. Beijing, Shanghai, and Tianjin all enjoyed an improving trend in decoupling state, but Chongqing had a deteriorating decoupling state. Moreover, Shanghai and Tianjin appeared to have a strong decoupling trend, indicating that the effects of transport’s carbon emissions in Shanghai and Tianjin were stronger than in Beijing, without prejudice to economic development. In regard to decomposed factors, the energy-saving effect significantly promoted transport’s economic growth to decouple from carbon emission in the four municipalities. The carbon-reduction effect usually promoted transport’s economic growth to weakly decouple from carbon emission, and appeared to show a remarkably strong decoupling trend in Shanghai, indicating that Shanghai had optimized energy use, using more low-carbon energy instead of carbon-intensive energy. The impacts of the economic scale’s effect on decoupling in Beijing, Shanghai, and Tianjin were better than that of Chongqing.

In response to our findings in this study, we suggest that it is urgent to transform the economic growth mode of the transport sector. Introduce foreign, advanced information technology to achieve logistics information sharing, and reduce unnecessary repetitive transport. In addition, rationally plan the transport network, build three-dimensional traffic, and improve traffic efficiency. In addition, adjust the transport mode of the transport sector and optimize the energy structure of vehicles. The state should introduce some relevant preferential policies, financial subsidies, tax incentives, etc.; eliminate transport vehicles with high energy consumption and high carbon emission; promote the development, promotion, and application of new energy and clean energy vehicles; and improve the construction of related support facilities. Continuously improve the proportion of clean energy used, such as solar energy, wind energy and biomass energy, and reduce the dependence of the transport sector on fossil energy. Finally, accelerate the research and development of energy-saving technology. Encourage relevant universities, institutions, and enterprises to establish technological innovation alliances to realize the study–research–production of energy-saving technologies. Increase investment in energy-saving technology’s development and use economic incentives to promote technological development. Actively cooperate with advanced countries with energy-saving technologies and strive to obtain the transfer of energy-saving technologies.

## Figures and Tables

**Table 1:** Table of various energy correlated coefficients.

Energy Type	Energy Conversion Coefficient (10^-6^TJ/Kg or m^3^)	Carbon Emission Factor (t/Kg)	Carbon Oxidation Rate
raw coal	20.908	25.8	0.90
coke	28.435	29.2	0.93
fuel oil	41.816	21.1	0.98
gasoline	42.070	18.9	0.98
kerosene	43.070	19.6	0.98
diesel	42.652	20.2	0.98
natural gas	38.931	15.3	0.99

**Table 2:** Decoupling decomposition results of Beijing’s transport sector.

Year	Individual Influencing Factors										Total	
	t_r_	State	t_s_	State	t_d_	State	t_f_	State	t_p_	State	t	State
2000–2001	0.12	WD	0.58	WD	0.12	WD	0.79	WD	0.13	WD	1.74	END
2001–2002	0.05	WD	–0.31	SD	0.13	WD	0.62	WD	0.24	WD	0.72	WD
2002–2003	–0.08	SD	–0.55	SD	–0.04	SD	0.78	WD	0.23	WD	0.35	WD
2003–2004	0.04	WD	1.36	END	–0.14	SD	0.97	EC	0.25	WD	2.48	END
2004–2005	0.02	WD	–0.58	SD	0.08	WD	0.65	WD	0.23	WD	0.40	WD
2005–2006	0.03	WD	0.73	WD	0.09	WD	0.64	WD	0.32	WD	1.81	END
2006–2007	0.02	WD	0.19	WD	0.03	WD	0.62	WD	0.36	WD	1.22	END
2007–2008	0.03	WD	0.16	WD	0.22	WD	0.28	WD	0.50	WD	1.20	END
2008–2009	0.02	WD	–0.61	SD	0.00	SD	0.48	WD	0.49	WD	0.38	WD
2009–2010	0.08	WD	–0.24	SD	–0.10	SD	0.50	WD	0.60	WD	0.83	EC
2010–2011	0.03	WD	–0.31	SD	0.07	WD	0.58	WD	0.34	WD	0.71	WD
2011–2012	–0.03	SD	–0.71	SD	0.02	WD	0.64	WD	0.31	WD	0.23	WD
2012–2013	0.06	WD	–0.29	SD	–0.01	SD	0.71	WD	0.30	WD	0.76	WD
2013–2014	–0.02	SD	–0.20	SD	0.03	WD	0.73	WD	0.24	WD	0.77	WD
2014–2015	0.07	WD	–0.72	SD	0.14	WD	0.72	WD	0.11	WD	0.32	WD
2015–2016	0.05	WD	–0.34	SD	0.03	WD	0.95	EC	0.01	WD	0.69	WD

END (expansive negative decoupling), EC (expansive coupling), WD (weak decoupling), and SD (strong decoupling).

**Table 3:** Decoupling decomposition results of Shanghai’s transport sector.

Year	Individual Influencing Factors										Total	
	t_r_	State	t_s_	State	t_d_	State	t_f_	State	t_p_	State	t	State
2000–2001	–0.06	SD	0.78	WD	–0.17	SD	0.75	WD	0.44	WD	1.75	END
2001–2002	0.01	WD	0.68	WD	–0.09	SD	0.83	EC	0.29	WD	1.72	END
2002–**–**2003	0.02	WD	1.00	EC	–0.46	SD	1.09	EC	0.41	WD	2.06	END
2003–2004	0.02	WD	1.31	END	–0.06	SD	0.80	EC	0.34	WD	2.41	END
2004–2005	0.02	WD	0.19	WD	0.00	WD	0.73	WD	0.28	WD	1.23	END
2005–2006	0.01	WD	0.44	WD	0.00	SD	0.68	WD	0.35	WD	1.48	END
2006–2007	0.00	WD	–0.20	SD	0.15	WD	0.52	WD	0.31	WD	0.78	WD
2007–2008	–0.01	SD	–0.69	SD	0.13	WD	0.50	WD	0.33	WD	0.25	WD
2008–2009	–0.01	SD	–0.87	SD	0.30	WD	0.39	WD	0.26	WD	0.07	WD
2009–2010	–0.01	SD	–0.06	SD	–0.77	SD	1.02	EC	0.74	WD	0.93	EC
2010–2011	–0.01	SD	–1.27	SD	0.13	WD	0.61	WD	0.19	WD	-0.34	SD
2011–2012	0.00	WD	–0.79	SD	0.27	WD	0.56	WD	0.13	WD	0.17	WD
2012–2013	–0.02	SD	–0.95	SD	0.12	WD	0.68	WD	0.17	WD	-0.01	SD
2013–2014	–0.01	SD	–0.98	SD	0.19	WD	0.72	WD	0.05	WD	-0.04	SD
2014–2015	–0.01	SD	–0.51	SD	0.33	WD	0.69	WD	-0.04	SD	0.46	WD
2015–2016	0.00	SD	0.17	WD	0.27	WD	0.71	WD	0.02	WD	1.18	EC

**Table 4:** Decoupling decomposition results of Tianjin’s transport sector.

Year	Individual Influencing Factors										Total	
	t_r_	State	t_s_	State	t_d_	State	t_f_	State	t_p_	State	t	State
2000–2001	–0.08	SD	–0.74	SD	–0.02	SD	0.95	EC	0.03	WD	0.13	WD
2001–2002	0.14	WD	–0.40	SD	–0.11	SD	1.07	EC	0.03	WD	0.72	WD
2002–2003	–0.35	SD	1.60	END	–0.25	SD	1.28	END	0.04	WD	2.32	END
2003–2004	0.13	WD	–2.42	SD	–0.28	SD	1.04	EC	0.10	WD	-1.43	SD
2004–2005	0.27	WD	0.42	WD	–0.25	SD	1.11	EC	0.17	WD	1.73	END
2005–2006	0.10	WD	–0.80	SD	–0.28	SD	0.96	EC	0.28	WD	0.26	WD
2006–2007	0.01	WD	–1.05	SD	–0.05	SD	0.73	WD	0.25	WD	-0.11	SD
2007–2008	–0.01	SD	–0.16	SD	–0.11	SD	0.72	WD	0.38	WD	0.82	EC
2008–2009	0.00	WD	–0.45	SD	–0.08	SD	0.75	WD	0.30	WD	0.52	WD
2009–2010	–0.10	SD	–0.25	SD	–0.20	SD	0.77	WD	0.41	WD	0.62	WD
2010–2011	0.05	WD	–0.63	SD	–0.10	SD	0.77	WD	0.30	WD	0.38	WD
2011–2012	–0.02	SD	–0.66	SD	–0.09	SD	0.71	WD	0.34	WD	0.27	WD
2012–2013	–0.10	SD	–2.22	SD	0.00	WD	0.56	WD	0.30	WD	-1.47	SD
2013–2014	0.01	WD	–0.42	SD	0.04	WD	0.65	WD	0.30	WD	0.57	WD
2014–2015	0.07	WD	–1.09	SD	0.03	WD	0.72	WD	0.20	WD	-0.07	SD
2015–2016	0.03	WD	–0.90	SD	0.08	WD	0.78	WD	0.10	WD	0.09	WD

**Table 5:** Decoupling decomposition results of Chongqing’s transport sector.

Year	Individual Influencing Factors										Total	
	t_r_	State	t_s_	State	t_d_	State	t_f_	State	t_p_	State	t	State
2000–2001	0.03	WD	–0.69	SD	0.01	WD	1.04	EC	–0.08	SD	0.31	WD
2001–2002	0.04	WD	–0.66	SD	–0.11	SD	1.14	EC	–0.06	SD	0.36	WD
2002–2003	0.04	WD	–0.66	SD	–0.21	SD	1.22	END	–0.04	SD	0.35	WD
2003–2004	0.94	EC	10.88	END	–0.25	SD	1.82	END	–0.05	SD	13.34	END
2004–2005	0.01	WD	–0.61	SD	0.06	WD	0.89	EC	0.01	WD	0.37	WD
2005–2006	0.01	WD	–0.44	SD	0.12	WD	0.83	EC	0.03	WD	0.54	WD
2006–2007	0.08	WD	0.75	WD	–0.25	SD	1.28	END	0.03	WD	1.88	END
2007–2008	0.00	WD	0.10	WD	–0.14	SD	1.08	EC	0.07	WD	1.11	EC
2008–2009	–0.06	SD	–1.36	SD	–0.09	SD	0.94	EC	0.05	WD	–0.51	SD
2009–2010	0.02	WD	0.71	WD	–0.37	SD	1.33	END	0.08	WD	1.78	END
2010–2011	–0.08	SD	–0.24	SD	–0.47	SD	1.34	END	0.11	WD	0.66	WD
2011–2012	–0.11	SD	0.44	WD	–0.13	SD	1.07	EC	0.08	WD	1.35	END
2012–2013	0.01	WD	–0.10	SD	–0.02	SD	0.94	EC	0.07	WD	0.91	EC
2013–2014	0.17	WD	–1.74	SD	–0.08	SD	0.93	EC	0.07	WD	–0.64	SD
2014–2015	0.05	WD	0.60	WD	0.04	WD	0.91	EC	0.08	WD	1.69	END
2015–2016	0.03	WD	–0.38	SD	0.03	WD	0.86	EC	0.10	WD	0.63	WD
